# Development of a Peer Support Mobile App and Web-Based Lesson for Adolescent Mental Health (Mind Your Mate): User-Centered Design Approach

**DOI:** 10.2196/36068

**Published:** 2022-05-27

**Authors:** Louise Birrell, Ainsley Furneaux-Bate, Jennifer Debenham, Sophia Spallek, Nicola Newton, Catherine Chapman

**Affiliations:** 1 The Matilda Centre for Research in Mental Health and Substance Use The University of Sydney Sydney Australia

**Keywords:** mobile health, depression, anxiety, psychosocial support system, alcohol drinking, adolescent, digital technology, mobile intervention, intervention, social, economic, development, mind your mate, app, application, mHealth, mobile phone

## Abstract

**Background:**

Digital technologies and mobile interventions are possible tools for prevention initiatives to target the substantial social and economic impacts that anxiety, mood, and substance use disorders have on young people.

**Objective:**

This paper described the design and development of the *Mind your Mate* program, a smartphone app and introductory classroom lesson enhancing peer support around the topics of anxiety, depression, and substance use for adolescents.

**Methods:**

The development of *Mind your Mate* was an iterative process conducted in collaboration with adolescents (n=23), experts, school staff, and software developers. The development process consisted of 3 stages: scoping; end-user consultations, including a web-based survey and 2 focus groups with 23 adolescents (mean age 15.9, SD 0.6 years); and app development and beta-testing.

**Results:**

This process resulted in a smartphone peer support app and introductory classroom lesson aimed at empowering adolescents to access evidence-based information and tools to better support peers regarding mental health and substance use–related issues. The program contains links to external support services and encourages adolescents to reach out for help if they are concerned about themselves or a friend.

**Conclusions:**

The *Mind your Mate* program was developed in collaboration with a number of key stakeholders in youth mental health, including adolescents. The resulting program has the potential to be taken to scale to aid prevention efforts for youth mental health and substance use. The next step is to conduct a randomized controlled trial testing the feasibility, acceptability, and efficacy of the program.

## Introduction

### Background

Mental health and substance use problems are common in the general population and increase across childhood and adolescence [1]. They are the leading cause of disability among young people worldwide [2], causing substantial impairment both during adolescence and later in life. Recent evidence indicates that mental health problems among youths are worsening, with data worldwide showing a historic rise in rates of depression and emotional problems among recent cohorts of youths, even before the COVID-19 pandemic [3-5]. Alongside access to treatment, effective and novel prevention efforts are needed to avert the costs and rising tide of poor mental health among youths. The use of web-based technologies and mobile health (mHealth) interventions provides a possible avenue for large-scale prevention initiatives.

Despite the impact and early onset of mental health and substance use problems, most young people do not seek professional help [6,7]. Two key barriers to help-seeking are stigma around accessing mental health care and an attitude of self-reliance [8,9]. When asking where they would turn for help, a large annual survey of Australian young people consistently found peers to be the number 1 reported source of support for young people in the previous 4 years [10-13]. Young people with mental health problems are also most likely to seek help from peers and family ahead of health professionals [14]. Peers play an important role during adolescence, helping shape identity as young people develop increasing independence from their family unit [15]. Peers are also uniquely positioned to notice behavioral or emotional changes in their friends, although many are unequipped to approach or support a friend showing early symptoms of mental health difficulties [16]. Therefore, it is important that young people are given the appropriate skills and knowledge to support peers regarding mental health and substance use in a format that is accessible and engaging to them. A systematic review of existing school-based peer interventions showed that, despite the widespread use of peer interventions, there is a lack of evidence demonstrating improvements in mental health and well-being [17], although 1 face-to-face adolescent peer-training mental health intervention (Teen Mental Health First Aid) demonstrated promising improvements in mental health literacy and intentions to seek help [18].

Increasingly, communication between adolescent peers is occurring on the web, and almost all adolescents in high-income countries now own or have access to a smartphone [19-21]. Adolescents report regularly using their phones to stay in touch with friends and access information [22]. Using smartphone devices and the internet to improve engagement and delivery of mental health care has been heralded as a potential avenue to take mental health prevention and early intervention programs to scale, particularly for the current generation of young people whose use of technology is ubiquitous in everyday life [23]. Over the past decade, there has been a proliferation of mobile app technologies for mental health. Most of these apps focus on self-help for the app user, such as tracking mood and using cognitive behavioral therapy or mindfulness techniques. However, the most commonly searched for mental health and substance use apps have limited evidence of effectiveness in improving mental health outcomes [24].

Although mental health apps have proliferated, there is a lack of evidence-based interventions aiming to upskill adolescents regarding supporting peers in relation to mental health and substance use. A literature review of mHealth peer support interventions showed a lack of research evaluating the use of apps in peer support for mental health [25]. When the search was broadened to include health outcomes more generally, it was found that, although youths have pre-existing digital comfort and benefit from peer support, most of the existing commercial and research-based health apps often encounter problems with engagement, which in turn hinders intervention efficacy [25]. A peer support app named the *Companion App* was evaluated in a control group study in Sweden [26]. It aimed to reduce unemployment-related stress among adolescents while fostering peer support through discussion groups. Although the adolescents endorsed the concept of the app, it had no effect on stress levels or the perception of social support, possibly because of a lack of engagement and inconsistent use of the app by the intervention participants during the trial [26]. It is well-recognized that involving end users of technology in the design process results in higher engagement and satisfaction levels [27-29]. For this reason, it is crucial to include the voices and perspectives of adolescents themselves alongside other key stakeholders from the start of the development process for novel web-based and app mental health interventions [28].

### Objective

In summary, despite very high levels of web-based communication among adolescents and the fact that peers are commonly the first port of call for young people experiencing mental health problems, there are currently no effective web-based interventions to assist adolescents to better support their peers regarding mental health. To address the need for an effective, evidence-based mobile intervention to assist adolescents in better supporting friends regarding mental health and substance use, we developed the *Mind your Mate* program. This paper describes the co-design process of the program, which consists of a classroom lesson plus a companion smartphone app.

## Methods

### Overview

**Figure 1:**
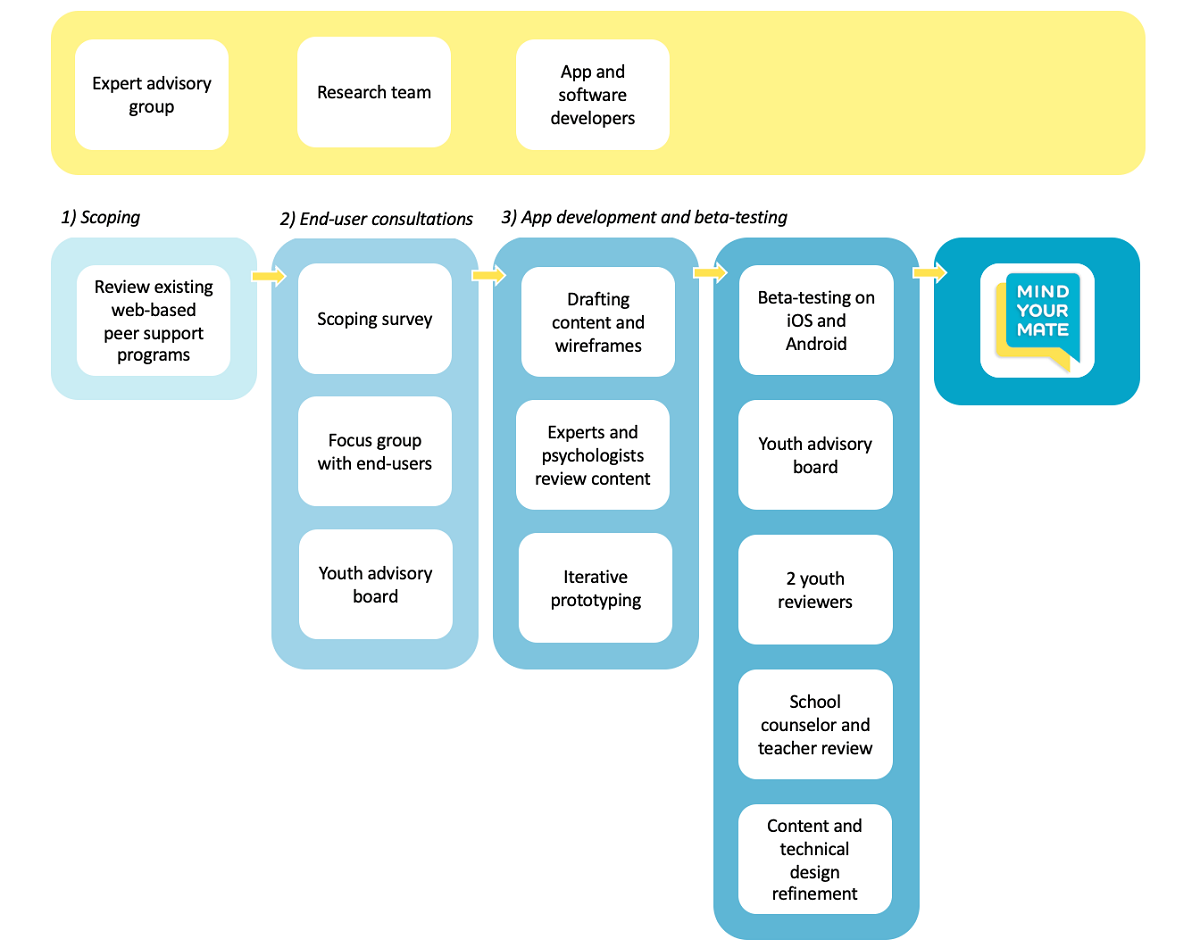
Overview of the *Mind your Mate* development process.

The development of *Mind your Mate* was an iterative process conducted in collaboration with adolescents, experts, and software developers. It drew on principles from the *create* phase of the accelerated creation-to-sustainment model framework [30]. As illustrated in Figure 1, the codevelopment process consisted of three stages: (1) scoping the existing app landscape, (2) end-user consultations, and (3) app development and beta-testing. The development process incorporated user-centered design principles [31].

### Stage 1: Scoping

As part of the formative process, we conducted a scoping review of existing peer support mobile apps, aiming to identify common features, functionality, and gaps. We searched for evidence-based peer support programs that focused on youth mental health or substance use across both the Apple App and Google Play stores. Search terms included “peer support,” “supporting friends’ mental health,” “friends’ wellbeing,” “youth mental health,” “wellbeing chats,” “friends’ wellbeing check,” “alcohol and drug wellbeing,” and “peer support for quitting.” Apps that fell under our search terms and were directed at youths were included in our scoping review (Table 1). The inclusion criteria were youths as the target audience and at least one of the following: mental health or alcohol and other drug education, focus on helping others, or interpersonal communication support (structured guidance for conversations with others and a forum for users). Owing to the limited number of youth peer support apps, we did not exclude self-help apps that met the criteria.

**Table 1:** Scoping review of existing peer support apps.

Included features	Selected apps					
	Chats for life	The Check-In	SAM: Self-help Anxiety Management	Stigma	Moodnotes	Woebot
Focus on helping others	✓	✓				
Self-help for carer						
Structured guidance for conversations with others	✓	✓				
Reminders to follow up with others		✓				
Password and privacy measures	✓		✓	✓	✓	✓
Crisis hotlines	✓	✓	✓	✓	✓	✓
Forum for users			✓	✓		
Safety plan or information for emergencies	✓		✓	✓	✓	✓
Customizable features						
Inclusion of mental health or AOD^a^ education	✓		✓		✓	✓

^a^AOD: alcohol and other drugs.

### Stage 2: End User Consultations

To ensure that the *Mind your Mate* program was appropriate and tailored to the needs of adolescents, a web-based scoping survey and a face-to-face focus group were conducted with the target audience (adolescents aged 15-16 years).

#### Participants and Procedure

Following the advertisement of the study through the researchers’ networks, 2 independent schools (one male-only and one female-only) in Sydney, New South Wales, Australia agreed to participate in a short web-based survey and in-person focus group. School-based recruitment was used for convenience and because of the implementation model using school delivery. Following permission from the school principal, 1 Year 10 class per school was invited to take part in a short web-based survey followed by a focus group conducted at their school facilitated by 2 experienced researchers (AFB and JD). Each focus group was conducted in a quiet classroom and lasted approximately 50 minutes. Both active parental consent and participant consent were obtained before the survey and focus groups. A semistructured format was used during the focus group sessions. The discussion guide used to structure the focus group sessions can be found in Multimedia Appendix 1.

In total, 23 high school students aged 15 to 17 years (mean 15.9, SD 0.6 years; 14/23, 61% female) from 2 independent schools completed a confidential web-based survey through a secure web application, Qualtrics (Qualtrics International Inc), hosted on the server of the University of Sydney. All students then participated in a focus group (14/23, 61% in one focus group and 9/23, 39% in the other). Feedback was sought about key components, usability, and design during the focus groups, and the discussions were recorded using an audio recorder. The participants were entered into one of 2 draws to win an Aus $50 (US $35.49) Prezee gift card as reimbursement for their participation.

#### Measures

The participants completed a 20-item web-based survey that contained multiple-choice, Likert-scale, and open-ended questions (for the full survey, see Multimedia Appendix 2). The participants were asked to indicate whether they owned a smartphone; if yes, they were then surveyed on their use of web-based mobile apps (eg, *what are the top five apps your regularly use on your phone?*). They were also asked about their preferences around accessing information to support their peers when they might be feeling anxious or low or using alcohol or other drugs. Finally, the participants were asked about their likelihood of using a mobile app on this topic.

#### Analysis

Descriptive analyses were performed on the quantitative questions from the scoping survey using SPSS (version 25; IBM Corp). The focus group recordings were transcribed verbatim by a research assistant (SS). For both the qualitative responses from the scoping survey and qualitative responses from the focus groups, general inductive analysis [32] was conducted by 3 members of the research team (SS, AFB, and LB), who independently coded segments of text from the responses or transcripts. Common subthemes were generated from segments of the text. The coders then collectively arranged the subthemes into primary themes to create higher-order explanatory thematic groupings. The coding categories were then simplified and merged into overarching themes capturing the key themes and subthemes. The 3 coders discussed any discrepancies until all coders agreed on the major themes. This analysis method was selected for its ability to identify patterns in qualitative data without reflecting researcher bias or familiarity with the subject matter [33].

A similar inductive thematic analysis approach was used to code the open-ended responses of the Qualtrics scoping survey that the focus group participants completed. Open-ended responses were coded by 2 independent coders (SS and LB), who were blind to one another’s coding. Discrepancies were resolved via consensus, and themes and subthemes were generated to capture participant responses. The framework was finalized with the assistance of visually organizing the responses on a digital whiteboard platform, Miro (Miro Corporation), and they are displayed in Multimedia Appendix 3.

### Stage 3: Program Development and Beta-Testing

The design and content of the *Mind your Mate* program were informed by the aforementioned scoping review, survey, and focus groups.

Mental health content was adapted from the Mental Health module of the Climate Schools and Our Futures mental health prevention program [34]. After the initial drafting and adaptation of the content by the lead researcher (LB), each section was independently reviewed by 2 experts in either the mental health or drug and alcohol prevention field (researchers with PhD qualifications) and 2 registered psychologists. These experts were recruited from the researchers’ networks. Revisions were made based on suggestions from the experts.

Throughout the development process, we twice consulted the Youth Advisory Board (YAB) of the National Health and Medical Research Council Centre of Research Excellence in Prevention and Early Intervention in Mental Illness and Substance Use. The YAB comprises a representative group of young people who provide comments and feedback on research drawing on personal experiences. During the initial consultation, the YAB provided feedback on the initial draft content and features and the program name, provided suggestions, and identified gaps in the initial program proposal. Second, they reviewed the early designs of the program app and were invited to review a beta version of the app, providing feedback and suggestions for improvements.

The first beta version of the app was scripted in both the iOS and Android operating systems. Once a working beta version of the app had been developed, it was reviewed by 2 end users (aged 16-17 years), a high school teacher and a school counselor over a period of approximately 2 weeks. The teacher and school counselor were selected from a convenience sample and provided perspectives on implementation within the school setting. These stakeholders were asked to rate what they did and did not like about the app, comment on the suitability of the app for Year 9 and 10 students, comment on what they would change, and report on any bugs or glitches. Revisions were made in response to end-user feedback to improve the app before its public release on the iTunes and Google Play stores. A further round of testing and refinement by the research team (lasting approximately 2 months) ensured that all changes were implemented on both the iOS and Android operating systems.

### Ethics Approval

This procedure was approved by the University of Sydney Human Research Ethics Committee (2019/723).

## Results

### Stage 1: Scoping Review Results

The most common features and content covered by existing peer support apps included information about mental health and substance use literacy, inclusion of privacy measures, crisis hotlines, safety plans, and user forums. Although the existing apps typically contained one or more of these features, most did not include all features, and none included self-help information, indicating gaps in knowledge that the *Mind your Mate* program sought to address (Table 1). Only *Chats for Life* [35] and *Check-In* [36] provided functions related to conversing with and helping others. The features of both apps included structured conversation templates, and *Check-In* also included reminders to conduct follow-up conversations. However, both apps lacked support for the app users themselves.

### Stage 2: End User Consultations

#### Focus Group Results

The thematic analysis identified key themes summarized under the 2 broad categories of *peer support* and *app functions and features*. This distinction differentiated between students’ personal experiences of peer support and their expectations of a mobile peer support tool.

Under the broad category of *peer support*, subthemes included (1) looking after yourself, (2) barriers to helping friends, and (3) helpful strategies for assisting friends.

Under the second overarching theme, *app functions and features*, seven subthemes emerged: (1) visual aspect of the app, (2) content, (3) functions of the app, (4) engagement, (5) user experience, (6) trust, and (7) dissemination (Table 2).

**Table 2:** Focus group transcript thematic analysis themes and subthemes.

Theme and subthemes			Examples
**Peer support**			
	Looking after yourself	Manage pressure, responsibility, and expectations for the person who is helping Tips for dealing with the fear of making things worse or saying the “wrong thing”	
	Barriers to helping friends	Concerns about respecting and overstepping boundaries Worry about lingering in the negatives by talking about it	
	Helpful strategies for assisting friends	Asking for advice. Confiding in a teacher or a close family member they trust Checking in, recognizing that it is a continuous process with follow-up required	
**App functions and features**			
	Visual aspect of the app	Visual content: videos, cartoons, media, and songs A wall of text leads to disengagement	
	Content	Understanding what is wrong with a friend leads to more confidence and comfort helping them Quick access to helplines Want suggestions on how and who to link their friend to for more support	
	Functions of the app	Link to other apps should be simple and easy for within-app efficiency (copy and paste text or send via Messenger) Personalize and customize the visuals (colors) Checklist	
	Engagement	A sense of achievement, reward, and competition from games is engaging Too many notifications can be annoying; no hassling to come back to the app but rather something that catches your attention Tone within the app should be engaging and empathetic	
	User experience	Privacy is important: not showing activity to friends, not needing location, and being able to transparently see and control privacy Participants disagreed about inclusion of a social networking component	
	Trust	Participants trusted lived experience, influencers, celebrities, and professionals (psychologists and researchers) Distrust in internet resources as they are nonspecific and outdated and may be wrong	
	Dissemination	Social media is where participants learn about mental health initiatives Reduce stigma with influencers and athletes Participants were more likely to google a problem than search the app store; the website should link to the app	

#### Key Implications for App Development

##### Looking After Yourself

Participants agreed that young people as supporting peers required specific guidance to manage pressure, responsibility, and expectations. The importance of self-care was also emphasized by participants citing the concerns they had about supporting their peers; for example, “it’s really hard not to feel like all the pressure is on you.” An entire educational module and functions of the app were dedicated to supporting the user in self-care. This included mood trackers and customizable self-care activity lists.

##### Barriers to Helping Friends

The participants indicated that they would try a variety of responses when concerned about a friend (such as providing distractions “to get it out of their mind,” giving their friend space, and stepping away to respect boundaries) but also cited key barriers. The participants acknowledged feeling worried about bringing up negative topics and uncertainty around how to start conversations. These concerns were specifically addressed in the educational modules within the *Mind your Mate* app and via a structured conversation planner built into the app to assist with starting and planning conversations with friends.

##### Helpful Strategies for Assisting Friends

The strategies that emerged were largely related to conversing with a friend, such as tips on how not to interrupt with your own stories. Recognizing changes in mood or behavior, which context is best for a conversation, and how to reach out to an adult were also discussed. Therefore, these topics were included in the app’s educational modules. In addition, recognizing that helping a friend is a continuous process led to app features that reinforced ongoing conversations, such as reminders and scheduling check-ins as functions available in the app.

##### Visual Aspect of the App

There was broad consensus among the focus group participants that paragraphs of written text would lead to disengagement. It was suggested that visual content (ie, infographics and videos) should be included. In response, recorded videos and animations were created for inclusion in the app and web-based lesson. The animations are consistent with a light and bright theme to visually destigmatize the app so that “people don’t feel negative having [the app] because of the way it’s set up.”

##### Content

Although some participants reported that they had received some mental health or substance use education before the focus group, many indicated that they would like to know more about “why it happens...the science behind it” as well as a clear pathway or action plan about what to do if they needed to assist a friend. The tone of the app content was positioned as relatively casual and approachable to assist users in gaining confidence when reaching out to friends. Education was presented as a source of empowerment throughout the modules, including quick links to specific topics if a user felt overwhelmed by the information or needed more focused support.

##### Functions of the App

To guide the user through the process of checking in with and following up on a friend, the participants indicated that checklists that can be edited or interacted with and how-to instructions would be beneficial. They also discussed functions that could improve engagement, such as customizing the color theme and adjusting notification settings—both of which were implemented in the final app.

##### Engagement

Several key concerns emerged regarding short- and long-term engagement with educational apps. Some participants indicated that they usually deleted an app when it was no longer useful, whereas others said that they would keep an app on their phone just in case. As such, gamification to increase the sense of achievement, as well as staggered release of content, was incorporated to promote deeper engagement with the app.

##### User Experience

Privacy emerged as a key concern, with most participants being in favor of passwords and transparent privacy policies. However, disagreement arose around creating a social networking aspect within the app; some participants expressed interest in chatting with peers in the app, whereas some were concerned about how these conversations might be monitored. An additional user experience concern for the participants was whether the app was straightforward to use. Disliked apps were described as follows: “The layout is confusing, everything is blue and white, it’s just hard to use.”

Therefore, iterative prototyping and beta-testing were conducted to facilitate the creation of an intuitive user experience design.

##### Trust

The participants indicated 2 sources of trust: professionals and public figures. They noted that “half the stuff on the internet you may not trust, it could be wrong.” Specifically, a lack of trust in many mental health apps indicates the importance of emphasizing the evidence-based nature of a program, including promoting the expertise of the developers. Public figures with lived experience were perceived as not only credible but also relevant to the user.

##### Dissemination

Public figures emerged again as a method of gaining young people’s attention and trust, particularly the potential use of celebrities on social media. The participants indicated that they often learned about mental health initiatives through advertisements and activism on social media. If they were unsure of an issue, they expressed that they were more likely to search for it on the web than to search in an app store, leading to the recommendation that there could be a companion *Mind your Mate* website hosting a clear call to action to download the app.

#### Scoping Survey Results

##### Scoping Survey Quantitative Results

Most participants (20/23, 87%; Table 3) indicated that having access to relevant information about going through *tough times* would be somewhat or very helpful. This agreement was similarly reflected when questioned about alcohol and drug information (18/23, 78%). Access to tips on how to help a friend was also well-supported and translated into support of participants being likely or very likely to access this information through an app (19/22, 86%). Interestingly, only 50% (11/22; Table 4) of the participants indicated that they thought it was likely that other young people their age would use the app; however, 68% (15/22; Table 4) were likely or very likely to recommend the app to a friend.

**Table 3:** Agreement with statements regarding the usefulness of using a peer support mobile app (N=23).

Statement	Response, n (%)				
	Very helpful	Somewhat helpful	Neither helpful nor unhelpful	Somewhat unhelpful	Very unhelpful
How helpful would it be to have access to more information about why young people sometimes go through tough times (eg, they might be feeling anxious, low, or not themselves)?	8 (35)	12 (52)	3 (13)	0 (0)	0 (0)
How helpful would it be to have access to more information about how many young people use alcohol and other drugs (including the effects this can have on them)?	7 (30)	11 (48)	4 (17)	1 (4)	0 (0)
Do you think it would be helpful to have access to tips about how to support a friend who might be feeling anxious, low, or not themselves (including examples of what to say or do)?	13 (57)	8 (35)	2 (9)	0 (0)	0 (0)
Do you think it would be helpful to have access to tips about how to support a friend who might be using or thinking about using alcohol or other drugs (including examples of what to say or do)?	8 (35)	14 (61)	0 (0)	1 (4)	0 (0)

**Table 4:** Agreement with statements regarding the likelihood of using a peer support mobile app (N=23).

Statement	Response, n (%)				
	Very likely	Likely	Unsure	Unlikely	Very unlikely
Would you access this kind of information via a mobile app?	3 (13)	16 (70)	2 (9)	2 (9)	0 (0)
How likely would you be to use an app like this?	1 (5)	14 (64)	2 (9)	5 (23)	0 (0)
How likely do you think it is that other people your age would use an app like this?	0 (0)	11 (50)	11 (50)	0 (0)	0 (0)
How likely are you to recommend an app like this to a friend?	4 (18)	11 (50)	5 (23)	2 (9)	0 (0)

##### Scoping Survey Qualitative Results

#### Overview

The participants wrote about both their favorite and challenging aspects of the mobile apps that they regularly used. Regarding peer support, the participants shared their needs when supporting friends and their preferences for information content and format. Each theme from these results is explained individually in the following sections, and a more detailed summary of the key themes can be found in Multimedia Appendix 4.

#### Favorite Aspect of Apps

The participants indicated that socializing is a well-liked aspect of their most frequently used apps. This included direct socialization, such as “I can talk to my friends,” as well as indirect socialization, such as “see how [my friends] are going and what they are doing.” Being able to intuitively use these apps also contributes to their place as favorites, with intuitive apps being described as “straight forward” and “smooth.” The enjoyment found in using these favorite apps was described as both entertainment and relaxation.

#### Unfavorable Experiences When Using Apps

Young people reported several unfavorable app features, including (1) interruptions of use such as glitches, advertisements, and time lags; (2) when an app consumes excessive resources (the participants indicated that their “phone battery drains very quickly when using TikTok” and that “addictive” apps can use up their data); and some participants reported (3) ambivalence about app use, including comments such as “they are a distraction sometimes” and “some apps [are]...harder to use.”

#### Helpful Resources When Worried About a Friend

The participants noted a variety of sources for *third party support*, such as counselors, parents, and “another person knowing what I knew about that friend.” Therefore, the content within the *Mind your Mate* program was designed to emphasize the importance of a support network as well as to encourage users to reach out to responsible adults and helplines. Another theme to emerge was *effective ways to help friends*, encompassing information about support methods and specific tips on how someone could support a friend who is struggling. In line with this theme, the need to know how to start a conversation emerged as its own specific concern. Fears of “trigger[ing] something,” uncertainties in how to approach a conversation, and wanting to “know helpful questions” indicated a broad need for assistance in talking to friends. The final *Mind your Mate* program provides scaffolds for conversations as well as checklists to prepare the user for potentially difficult discussions.

#### What Kind of Information

When asked what kind of information would be useful, the participants’ answers fell under the theme *how to have a conversation*. Many had difficult questions that they wanted to ask their friends, such as “why do they use alcohol and drugs?” and “are they being influenced?”. Participants wanted more information about how to ask these questions, as well as how best to respond. They also indicated that they wanted information to discourage substance use, including “evidence and statistics,” as well as ways to “remind them of the consequences.” Therefore, the program content includes educational modules on these topics. *Mind your Mate* also provides extensive resources for users to reach out to a third party as the participants indicated that they wanted to know “where to go, who to tell [and] what to tell them.”

#### Information Formats

Most participants (19/23, 83%) indicated that they would access information about supporting friends in the form of an app, whereas almost half (11/23, 48%) reported they would also like to see this information presented via a website. A student said the following: “An app would be an ideal format, but a website would be helpful as well.”

Other suggestions included offline visual media, such as a brochure, and web-based visual media, such as YouTube and Instagram posts. In addition, verbal presentations were suggested in the form of workshops, seminars, and school talks. The participants were supportive of the idea of developing an app.

### Stage 3: Program Development and Beta-Testing

#### Overview

Following the user-centered design process, a beta version of the *Mind your Mate* mobile app and web-based classroom lesson was created. End-user consultations and beta-testing of the app and classroom lesson were conducted, and minor updates were made to the app in response. A logic model underpinning the program can be found in Multimedia Appendix 5.

#### Web-Based Classroom Lesson

One of the most common barriers to mHealth programs are uptake and engagement with the intervention [37]. This is important as higher levels of engagement with digital programs are associated with improved mental health outcomes [38]. Schools represent an ideal delivery avenue to enhance uptake and engagement with mHealth prevention programs, providing large numbers of adolescents with access to mental health content linked to existing well-being curricula. To aid in the uptake and dissemination of *Mind your Mate*, we planned an implementation model using delivery through high schools, designing a classroom lesson (introducing the concept of mental health literacy and supporting friends) with students downloading and registering on the app at the end of the lesson. This lesson was integrated into regular schooling and linked to the existing curriculum on drug and alcohol use and mental health. This model enabled *Mind your Mate* to be linked to the existing school system and delivered to whole school cohorts, thereby reaching large numbers of adolescents who may not otherwise engage with the app if it was listed on the app stores only. The implementation and sustainability of the program was thought to be enhanced if the intervention had the capability to be embedded into existing structures and systems. This approach was also seen as favorable in overcoming potential biases introduced when digital intervention recruits highly selected samples from potentially large pools of web-based participants (the denominator problem [30]). In prevention, the education and school system are the main contact points for adolescents, in contrast to treatment services and the health care system for treatment interventions. Findings from the digital treatment literature also show enhanced outcomes when human support is included alongside digital technologies [39]; hence, teachers were used to facilitate the web-based lesson and ensure the download of the app, although this lesson could be facilitated by any school staff member.

**Figure 2:**
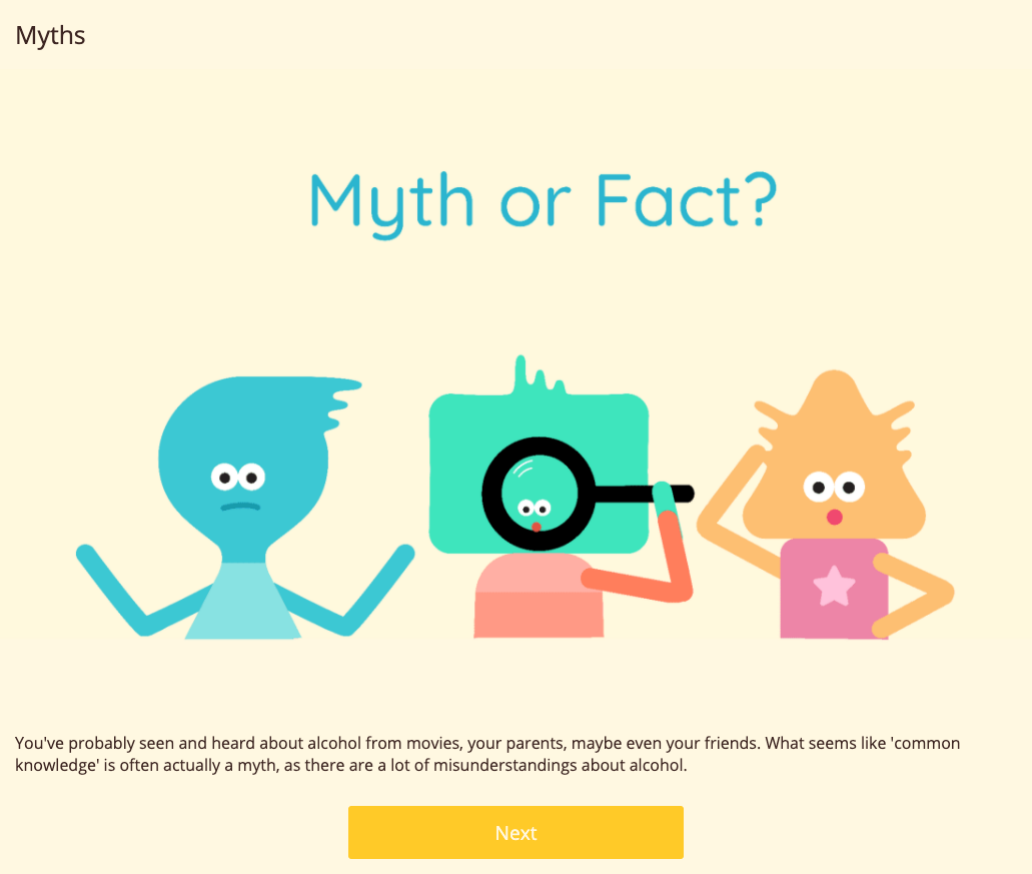
Selected screenshot of the *Mind your Mate* web-based lesson.

On the basis of teacher feedback (that student engagement might benefit from an introduction to the app in class) and the aforementioned rationale, we developed a short introductory lesson (approximately 40 minutes long) accessed through a web-based portal (see Figure 2 for screenshots). The lesson’s subject matter was derived from content prepared by the lead researcher (LB) and experts, with a focus on normative challenging and active listening techniques. During this lesson, students watch a series of explanatory and narrative short videos, take part in interactive quizzes and classroom discussions, and role-play activities. They are introduced to core concepts of mental health literacy, alcohol use, and peer support. At the end of the lesson, all students are prompted to download the smartphone app and shown a short introductory explainer video on how to use and navigate the app.

#### Mobile App

**Figure 3:**
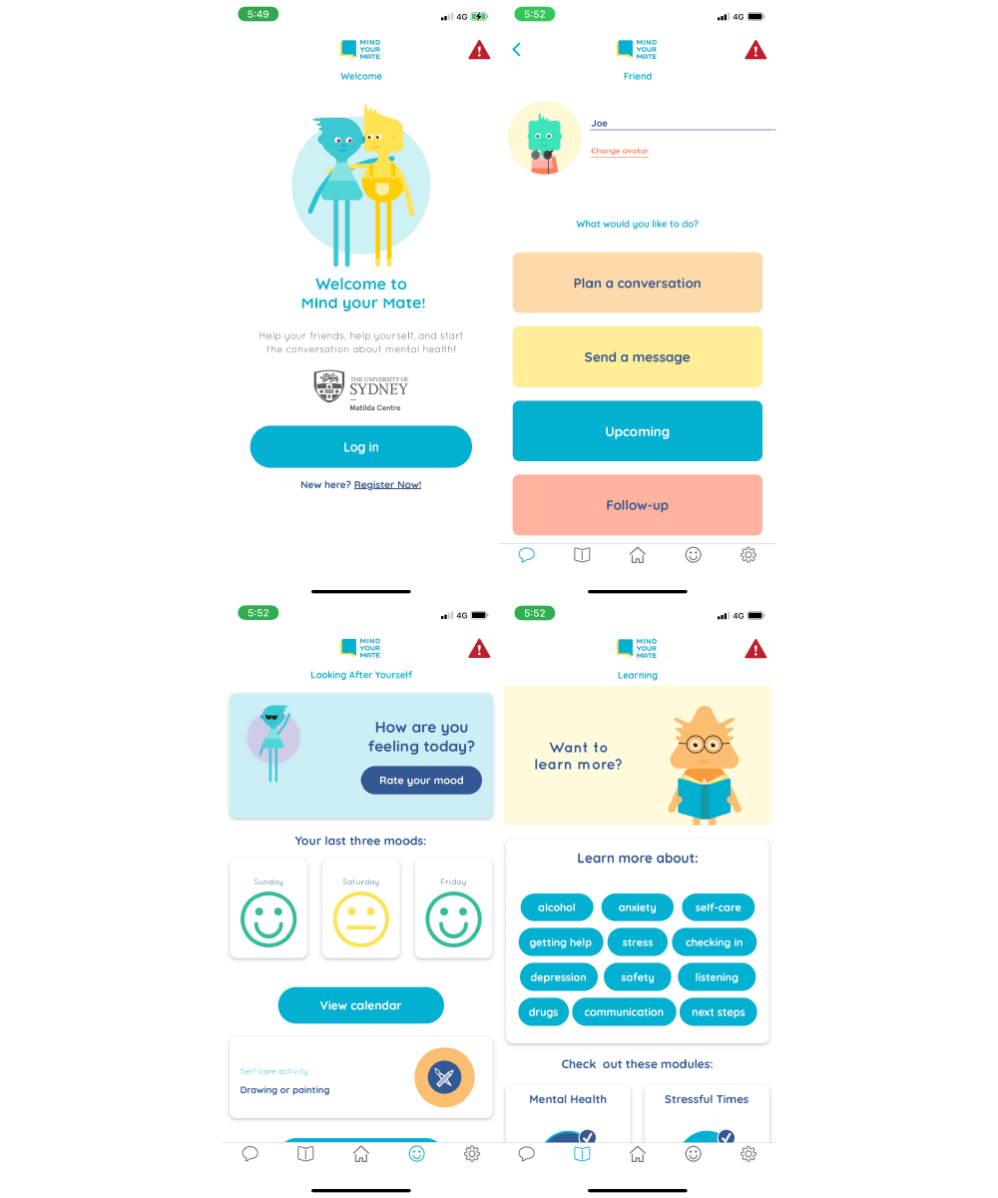
Selected screenshots of the *Mind your Mate* app.

The mobile app included the following key sections—*Friends*, *Learning*, *Home*, *Self-care*, and *Settings*—displayed as separate tabs within the app (see Figure 3 for screenshots). Table 5 also summarizes the key components of the final app, which include conversation scaffolds and checklists to build users’ confidence in discussing mental health and substance use, reminders to conduct follow-up conversations and further the support given to friends, accessible mental health and substance use literacy content, and tracking the user’s own mood. The app also includes a button to access helplines, the Australian emergency number, Lifeline, and KidsHelpLine, which are always visible and accessible on every page.

In the *Friends* section, users can create custom profiles for their friends. They are prompted to plan a conversation, send a message, view scheduled conversations that are upcoming, and follow up on past conversations. The conversation planner helps the user scaffold a conversation with their friend, prepares them, and adds the conversation to their phone’s calendar. A similar scaffold helps users review and follow up after a conversation has taken place. Example messages can be edited and copied and pasted from the app onto other messaging platforms, not only helping users start conversations but also creating an optional function for socializing.

The *Learning* section contains the educational modules with quick links to specific topics for easy access. Content is provided in paragraph, dot-point, infographic, animation, and video format. The module icons track the user’s progress and award a tick mark after a module has been completed.

The *Home* section features a greeting to the user with the customized avatar. It also presents quick links to educational modules, a reminder of upcoming conversations, and a mood rating function. The landing page redirects the user to various functions of the app.

The *Self-care* section relates to looking after oneself, where users can rate their mood. The most recent mood ratings are displayed, and a broader history can be accessed by viewing a calendar. This allows users to maintain an oversight of their own well-being. To aid a user if they themselves are struggling with their well-being while helping a friend, a curated list of self-care activities can be created. Users can select from a variety of suggested healthy activities as well as write their own custom activities.

In the *Settings*, users can edit their profile information and notification settings. To foster trust in the app and promote its evidence-based nature, the privacy policy and a section about *Mind your Mate* are also available for reading. Users may also customize their experience within the *Settings*. Here, users can select their avatar’s shape, color, and accessories. A name and hobbies can also be selected as well as picking between 2 color themes for the app overall. Personalization was often mentioned throughout the co-design process as an important engagement factor.

**Table 5:** Overview of the *Mind your Mate* smartphone app educational module content.

Module name	Key content covered	Related features
Mental health	Anxiety and depression literacy	Animated videos featuring personal stories Symptom diagrams Myth buster
Stressful times	Managing well-being References to COVID-19 and bushfires	Links to trusted external resources for self-help and emergencies
Alcohol and drugs	Substance use literacy, normative use, and standard drinks	Animated videos Symptom diagram Quiz question External link to standard drink calculator
Listen up	Active listening skills Tips and tools to start conversations with friends	Scheduling conversations that link directly to the calendar Example text to send to friends
Keeping friends safe	Harm minimization for substance use, supporting disclosure to parents or professionals	Recovery position diagram Video challenging fears of responsibility for a mate Harm minimization and emergency response video
Tricky conversations	Active questioning, motivational interviewing, and communication skills	Conversation preparation checklist and template Template messages to send to friends when reaching out Emotion dictionary
Checking in	Following up with friends, conversation tips	Scheduling conversations to follow up directly into calendar Support links Profiles for friends to reference upcoming and past conversations
What next?	Challenges notion of having sole responsibility for another’s well-being, supporting disclosure to parents or professionals	Template to review conversation with a friend Tick-box checklist Textbox for reflection
Support options	Provision of formal and informal support options such as school counselor, parent, free web-based resources, and general practitioners	Template messages to send to friends when reaching out Links to trusted external educational sites, resources, and support
Looking after yourself	Self-care Emotion regulation skills	Personalization of self-care activity list Ability to add custom activity Mood tracker

## Discussion

### Principal Findings

This study outlines the co-design process of *Mind your Mate,* an mHealth app developed to assist young people to better support their friends regarding mental health and substance use. The iterative, user-centered design approach included peer and expert reviews at all stages of development, from initial scoping to prototyping and beta-testing. Most of the adolescents surveyed reported that they would find it helpful to have access to more information about mental health (21/23, 91%) and alcohol and drugs (22/23, 96%) and that they would access this information in the form of a mobile app (19/23, 83%). The mHealth app aims to empower young people to develop action plans, set goals, and track progress through access to comprehensive, evidence-based information and strategies on mental health and substance use.

The initial scoping review showed that most existing peer support mHealth apps include the provision of comprehensive mental health and substance use information, access to crisis hotlines, safety plans, and strict privacy measures. The scoping review also illuminated several gaps in mHealth apps, particularly regarding the lack of evidence of effectiveness, which is consistent with a recent systematic review of mHealth apps in mental health [40] and substance use [41]. In addition, currently no peer support app provides information on self-help for the carer despite this being essential in developing adolescent self-regulation and health promotion skills [42]. The importance of self-help strategies was explored and confirmed during end-user consultations, whereby adolescents identified self-help as important in combating burnout and fear of responsibility when supporting a friend. Indeed, other studies have found that self-care is lacking in adolescent mental health, substance use prevention, and health promotion [40]. There is evidently a large need for further self-help strategies and information to be delivered to adolescents when substance use and mental health problems begin to emerge.

A significant barrier to the impact of mHealth apps is their low initial uptake and decreasing engagement over time [43]. Consultations with key stakeholders such as teachers and a school counselor informed the innovative 2-part implementation of *Mind your Mate* whereby students are introduced to peer support and self-help via a web-based portal during class time and then prompted to download and explore the companion mobile app. This implementation strategy aims to help break down known barriers to adolescent engagement with mental health and substance use mHealth apps and ensures that the critical and key components of the intervention are delivered to all students within the classroom setting. Consultations with adolescents highlighted the role that celebrities and public figures can play to increase engagement with apps by building trust and credibility in the first instance, and this dissemination strategy has been successfully applied in health areas [44]. Several additional strategies arose to help promote sustained engagement with the app. This included practical tools and strategies such as structured conversation prompts—which few current apps include—customization through color schemes and avatars, gamification through progress levels and rewards, and the frequent release of new content updates.

During end-user consultations, privacy emerged as a key concern, with adolescents requesting passwords and transparent privacy policies. Notably, there were mixed reviews of social networking features in mHealth apps. Although many adolescents reported socializing both actively (messaging) and passively (public posts and activity status) to be a favorite element of their most used apps, focus groups revealed that students worry about breaching their friends’ privacy by discussing their concerns with a parent or counselor. By extension, it was determined that students’ engagement with the app’s core functions may be hindered rather than supported by communication and sharing between users. Thus, because of accentuated privacy concerns around mental health and substance use and the risk of irresponsible socializing, social networking functions were not built into the *Mind your Mate* app.

### Limitations

The results of this study must be interpreted within the constraints of several limitations. First, the initial scoping review of currently available apps was not a formal systematic review. Nonetheless, the app was designed with well-proven peer support and self-care strategies that help promote well-being for adolescents (goal setting, action plans, and self-monitoring). Second, although end users identified celebrities and public figures as important to increase engagement with the app, resourcing constraints during the first wave of the COVID-19 pandemic hindered this content from becoming a part of the first release of the app. However, a list of influential people to contact was collated, and these features are planned to be included in future iterations of the app. Finally, the iterative development process was informed by several key stakeholder groups whose sampling can be improved upon in future studies. For instance, both schools that participated in the focus groups were same-sex, which may have left a gap in insights on mixed-sex peer relationships. The relatively small sample size also means that the findings from the focus groups and survey should be viewed cautiously as early insights, and larger sample sizes with young people are required to pilot and evaluate the effectiveness of the app and inform future iterations. A key strength of this study is the user-centered design approach whereby end users, key stakeholders, and content area experts were involved at different stages of the development process. A critical next step in the development of the evidence base for *Mind your Mate* will be to conduct a large-scale randomized controlled trial of the effectiveness of the mHealth app in the target group of adolescents.

### Conclusions

Mental health and substance use carry significant and escalating harms for young people, and the burden can fall largely on peers during adolescence. Adolescence is the typical age of onset of mental health and substance use issues, and there is a great opportunity for peers to play a key role in supporting each other. The *Mind your Mate* mHealth app was developed to address the needs of young people to better support their peers regarding substance use and mental health and uniquely includes self-care strategies, gamified continuous support, and customizable content. A larger controlled trial currently underway is the next critical step to build the evidence base in this important mental health prevention area.

## Appendix

Multimedia Appendix 1 Discussion guide used to structure focus group sessions.

Multimedia Appendix 2 Web-based scoping questionnaire on app use for focus group participants.

Multimedia Appendix 3 Visual organization of open-ended scoping survey responses.

Multimedia Appendix 4 Scoping survey themes.

Multimedia Appendix 5 Logic model for the *Mind your Mate* app.

## Abbreviations

mHealth: mobile health
YAB: Youth Advisory Board
