# Supplementary material for: Development of a Peer Support Mobile App and Web-Based Lesson for Adolescent Mental Health (Mind Your Mate): User-Centered Design Approach
Source: JMIR Form Res. 2022 May 27;6(5):e36068. doi: 10.2196/36068 (PMC9187963; doi:10.2196/36068)
Supplement: Multimedia Appendix 1 [file formative_v6i5e36068_app1.pdf]

Verbal Focus Group Questions (Focus Group 1 and 2)

General App questions

1. **What apps do you use that you really like?**
   - **What are some of your favourite things about those apps?**
2. **Can you think of an app that you didn't like?**
   - **What was it about the app that you didn't like?**

We are now going to have a discussion about how people your age support their friends when their friends are going through a tough time. This is going to be a general discussion, and we will ask that you don't name anyone in particular. Most people will have seen their friends go through a tough time at some point. If you can't think of a certain situation or would prefer not to share any details it might help to think about what could be helpful if someone your age was trying to support a friend who was having a tough time.

Remember you don't have to share anything you don't feel comfortable with and if you don't want to take part at all, that's fine, just let us know and we will organise another activity for you.

Supporting friends going through tough times (content of app)

3. **Think about a time you were worried about a friend, could you tell me about what would have been helpful at that time?**
   (When I say you might have been worried about a friend, I mean they may have been feeling anxious, low, not themselves, or were thinking about using alcohol or other drugs)

We have just spoken about some topics that might have made you concerned for a friend or yourself. If any of the discussion made you feel uncomfortable, sad or worried, there are people you can reach out to. These could be your family, doctor, teachers or counsellors (e.g. school counsellors). At the end of the session we will hand out a slip to everyone with some more information to follow-up if you would like to.

Feedback on idea of app

4. **What do you think about the idea of using an app to help young people support their friends going through tough times?**
   Prompt: What would be good about it?
   Prompt: What would be not so good about it?
5. **Can you tell me about whether you would use an app like this, and describe why or why not?**
6. **In what circumstances would you feel like you would like to use this app?**
   Prompt: Would you use it for a friend you are a little bit worried about, or for a friend you are worried about a lot?
7. **Can you tell me about whether other people your age would use an app like this, and describe why or why not?**
