# Supplementary material for: Development of a Peer Support Mobile App and Web-Based Lesson for Adolescent Mental Health (Mind Your Mate): User-Centered Design Approach
Source: JMIR Form Res. 2022 May 27;6(5):e36068. doi: 10.2196/36068 (PMC9187963; doi:10.2196/36068)
Supplement: Multimedia Appendix 2 [file formative_v6i5e36068_app2.pdf]

Individual Questionnaire

1. **How old are you today? (years/months) _______________**

2. **Which of the following best describes your current gender identity? (tick box)**
   - ☐ Male
   - ☐ Female
   - ☐ Non-binary/gender fluid
   - ☐ Different identity: ____________

3. Do you own a smartphone (please circle)?

Yes	No

General app questions

4. What are the top five apps you regularly use on your phone?
   1.
   2.
   3.
   4.
   5.

5. What are some of your favourite things about those Apps?

____

____

____

____

6. How often do you use those Apps (on average)?

Every day or nearly every day	At least once a week	At least once a month	At least once a year	Never

7. What are some of the annoying things you experience when using apps?

____

____

____

Supporting friends

8. Have you ever been worried about a friend but were unsure what to say or do?

Yes	No

9. Have you ever googled how to help a friend?

Yes	No

10. Think about a time you were worried about a friend, what would have been helpful at that time?

11. How helpful would it be to have access to more information about why young people sometimes go through tough times and might be feeling anxious, low or not themselves?

Very helpful	Somewhat helpful	Neither helpful or unhelpful	Somewhat unhelpful	Very unhelpful

12. How helpful would it be to have more information about how many young people use alcohol and other drugs and the effects it can have on them?

Very helpful	Somewhat helpful	Neither helpful or unhelpful	Somewhat unhelpful	Very unhelpful

13. Do you think it would be helpful to have access to tips about how to support a friend who might be feeling anxious, low or not themselves (including examples of what to say or do)?

Very helpful	Somewhat helpful	Neither helpful or unhelpful	Somewhat unhelpful	Very unhelpful

14. Do you think it would be helpful to have access to tips about how to support a friend who might be using or thinking about using alcohol or other drugs (including examples of what to say or do)?

Very helpful	Somewhat helpful	Neither helpful or unhelpful	Somewhat unhelpful	Very unhelpful

15. What kind of information would you need if you were worried about a friend who was using or thinking about using alcohol or other drugs?

____

____

____

____

16. Would you access this kind of information via a mobile app?

Very likely	Likely	Unsure	Unlikely	Very unlikely

17. Are there any other formats would you like this information to come in?

____

____

Imagine there was an app available to download from the app store that had information, tips and support options to help you to support your friend when they might be going through a tough time (for example, they might be feeling anxious, low or you might be worried about them using alcohol or other drugs).

18. How likely would you be to use an app like this?

Very likely	Likely	Unsure	Unlikely	Very unlikely

19. How likely do you think it is that other people your age would use an app like this?

Very likely	Likely	Unsure	Unlikely	Very unlikely

20. How likely are you to recommend an app like this to a friend?

Very likely	Likely	Unsure	Unlikely	Very unlikely

Thank you for completing this questionnaire!

If any of the topics raised in this survey have made you feel uncomfortable, sad or worried, there are people you can reach out to. These could be your friends, family, doctor, teachers or counsellors (e.g. school counsellors). You can also phone:

Kids Helpline on 1800 55 1800 for free, confidential help with any issues you want to talk about (www.kidshelpline.com.au) or Lifeline on 13 11 14 for 24/7 crisis support (www.lifeline.org.au).
