# Supplementary material for: Development of a Peer Support Mobile App and Web-Based Lesson for Adolescent Mental Health (Mind Your Mate): User-Centered Design Approach
Source: JMIR Form Res. 2022 May 27;6(5):e36068. doi: 10.2196/36068 (PMC9187963; doi:10.2196/36068)
Supplement: Multimedia Appendix 3 [file formative_v6i5e36068_app3.pdf]

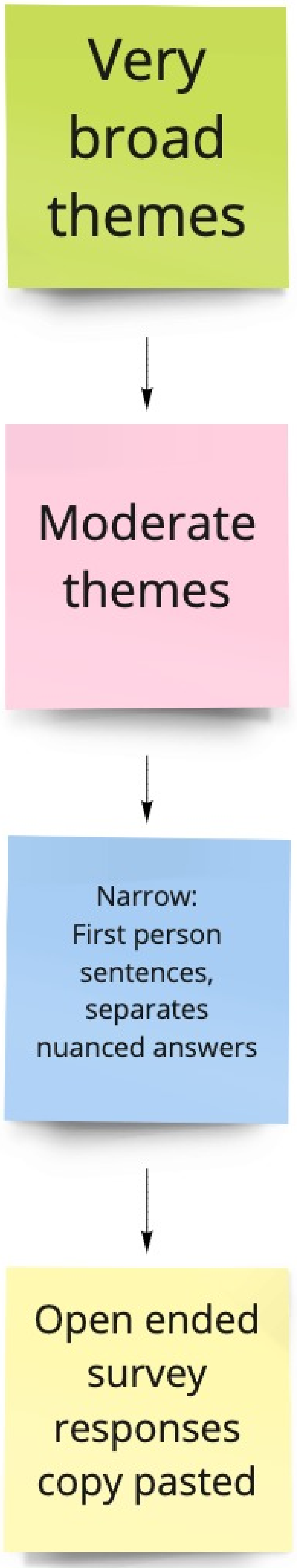
Very broad themes
Moderate themes
Narrow: First person sentences, separates nuanced answers
Open ended survey responses copy pasted

Socialising

Socialising

Indirect socialising: These apps let me see what others are up to.

Can listen to music, talk to people, see what other people are doing.

My friends are on it so I can see what they are up to. Very addictive game.

Everyone's using it

it helps me connect and communicate with other people when I am not around them.

Direct socialising: These apps let me interact with others.

Talking to my friends Staying incontact

My favourite things about these apps is that you able to check in on your mates and see how they are going and what they are doing. Also I like to listen to music.

You can interact with people

Fun to spend time on, allows me to interact with other people

I'm able to communicate with my friends that are either in a different state or country. Some apps just let me enjoy and relax

With snapchat you can talk to mates whenever. Facebook you can scroll through content whenever. Instagram you can see whaat friends have been doing by what they post. Messenger chats to mates whenever. TikTok look at random people's videos.

Enjoyable

Entertainment

These apps entertain me.

relatable content, make me laugh, contact friends, listening to songs help me cope with things that are going on with my life, able to relax

I love tiktok because I understand the humour and it is easy digestible content that makes me laugh. the community is unlike other and is very funny content. I like spotify because I can customise my taste and find things I would never normally listen to. I like messenger cause I can talk to my friends

they are social, keep me entertained

They provide entertainment (e.g. Instagram, YouTube, Spotify, Safari) and social connection (e.g. Instagram, Messages).

Watch others' lives, videos about games and sports and stuff.

I'm am able to communicate with my friends and also it's also a good source of entertainment when I am alone.

They provide a wide array of content, including entertainment (first four apps), fun education (from YouTube), and convenience (WhatsApp messaging and Bible app).

Relaxtion

These apps help me relax.

They allow me to talk to friends and let me see content that is posted by other users. I can also listen to music which helps me relax.

Easy to use, they have enjoyable purpose, they help me relax.

stay connected with friends, relatable content, makes me laugh, listening to songs helps me stay calm and focused especially when going through struggles, reading allows me to expand my imagination.

Intuitive use

Intuitive use

These apps are intuitive to use.

How simple and straight forward they are. The fact the app is free and I can use it with my friends.

chrome and samsung internet: they use generally the same icons across both apps to mean the same thing (e.g. '+' is used to indicate creating a new tab) YouTube: that it has both generally popular videos and videos that are catered towards me phone: simple categories (e.g. contacts, dial...) making it easy to understand

The accessibility that they give you to others and online resources, how smooth and well developed the technology is.

miro

Interruptions to use

Advertisement interruptions

Ads are annoying.

Ads

When games send you stuff that you don't care about. They also come up with ads which is annoying.

pop up ads or ads in general.

Some of the ads that I face when using the app.

Advertisements, angry commenters

Inability to use

Glitches are annoying.

Phone glitching out

when they crash, on the YouTube app when you can't have multiple videos open at the same time

Like when you're going for your highest score then you die. Sometimes snapchat just logs out randomly.

Sometimes the app crashes/lags, or glitches.

Sometimes it lags or glitches which can be annoying to fix.

Glitches out, music doesn't download, new look updates.

Uncertainty in use

Uncertainty in use

Uncertainty in how to use the app is annoying.

Sometimes these apps can provide us with unnecessary information. Also some apps take are 4 longer and harder to use.

They might crash, or glitch and they sometimes are not clear in how a process should be done, not being able to do simple tasks such as log out.

They are distractions sometimes

Requires resources

Requires resources

Apps that strain internet/data connection are annoying.

When the wifi isn't working properly so the app lags.

Glitching, being really slow, Ads

Bugs, INternet too slow

Glitches, Ads, when the apps start to slow down.

ads, needing to pay money, functions don't work unless you pay, uses heaps of data

Apps that require require battery power or keep me on them long enough to drain battery are annoying.

my phone battery drains VERY quickly when using tik Tok and the battery life has decreased I don't like how some apps don't have a dark mode

they are addicting therefore using up my data.

sometimes can be glitching, uses up battery life.

glitches, how long I spend on the app, battery loss

miro

Third party support

How to have a conversation

Best ways to help

Third party support

How to have a conversation

How to help

Offering help.

Professionals are helpful.

- Seeing a counsellor to get professional help/analysis on the friend's issue based on their symptoms and actions.
- Someone telling me that it isn't always entirely my responsibility to help them and being able to reach out to other people who have more experience or knowledge than me and are probably better equiped to help them.

Someone who knows my friend is helpful.

- someone to give me advice when the situation is occuring
- Another person knowing what I knew about that friend.
- To pray for them and maybe to have spoken to their parents about what was going on.
- Someone responsible and understanding of the situation to be able to talk it out with
- an adult, a parent. Time to get away and distract them from the problem.

I'm unsure of HOW to approach my friend.

- I think it would've been helpful to know what the appropriate thing to say to them was and other ways that I could help and support them.
- if I had known different ways to comfort them or be able to have the knowledge on what I can do
- To know helpful questions and ways to talk to them.
- It would be helpful if someone could tell me how I should approach my friend because I don't really know whether if I will say something that may trigger something and make the situation worse.

I'm unsure of WHAT to say to my friend.

- basic methods on how to help them or someone who was giving advice and helping me to help them.
- Clear, useful tips
- Support- making sure they were ok
- That I should've confronted them about the problem and how they're acting a bit off and whether they're truly okay even when they deny it.
- duke of ed is always a stressful time and many panic attacks and breakdowns occur and I think looking back I should have talked to my friend in a manner that was calming and approached the situation with some forethought into what they were going through
- One time one of my friends wasn't talking to me as much so I was worried. Something that would of help was checking in on them and seeing how they were going.

My plan would be to talk to my friend.

- If I went up to him and asked if he was alright. I could asked if there was anything I could do to help.
- Reaching out and asking whether they were okay or not, and if there was anything I could do to help them move through it.
- To go up to him and just say I'm here if you need to talk.

miro

How to have a conversation

Information to discourage use

Third party support

I DO NOT KNOW

How to have a conversation

I would want to ask about their reasons for use.

- How long they have started to use alcohol or drugs - How did they first start, was it peer pressure, curiosity - Why do they use alcohol and drugs and for what reasoning

His reasons and purposes.

- What made them want to use alcohol or other drugs - Why they started to think of drinking alcohol - What can I do to stop them from thinking like that

Where they are and what their doing.

What are the reasons they are using drugs or alcohol Are they being influenced

how to help them, what to say

Facts

I would like to know factual information.

Evidence and statistics to look at the situation logically - e.g. the associated health risks, side effects, deaths from substance abuse, any potential benefits. It would also be useful to know how to start the conversation and talk to the friend in a loving but realistic manner.

I'd need information about the effects of alcohol/drugs and any statistics that are relevant to the use of alcohol/drugs by teens.

I would like to know the effects on what that drug would do to them and if it would effect them in a good or a bad way.

the health effects of these personal stories relevant statistics

I would want to know how to talk about the consequences.

how to discourage them and remind them of the consequences.

How we could help the friend. How to stop them drinking. How to recognise.

What to say to them or how to help prevent them from doing anything that may harm them a fair bit.

I think it is important to tell people not to drink or do drugs but inevitably people are gonna do it they may know the repercussions but do it any way but I think it should be taught what not to do like drink red bull and alcohol because you can have a seizure

Cessation information

I would want to know how to talk about future prevention.

How to support them and help them make better decisions/find ways to stop using

how to talk them out of it, how to help them overcome the struggle that they are facing so they can not take the substances.

How to get them out of the mindset of wanting to use these

Like how you could distract them from not doing it, like an activity.

how to stop their urge to drink or do drugs

Third party support

I would like to know who to go to if it's not my place to say something.

How to help, treat them and help them if their drinking is not drastically out of control. Also who to contact if it is drastically out of control.

Where to go, who to tell, what to tell them

How do you convince them not to without damaging your relationship with them.

miro

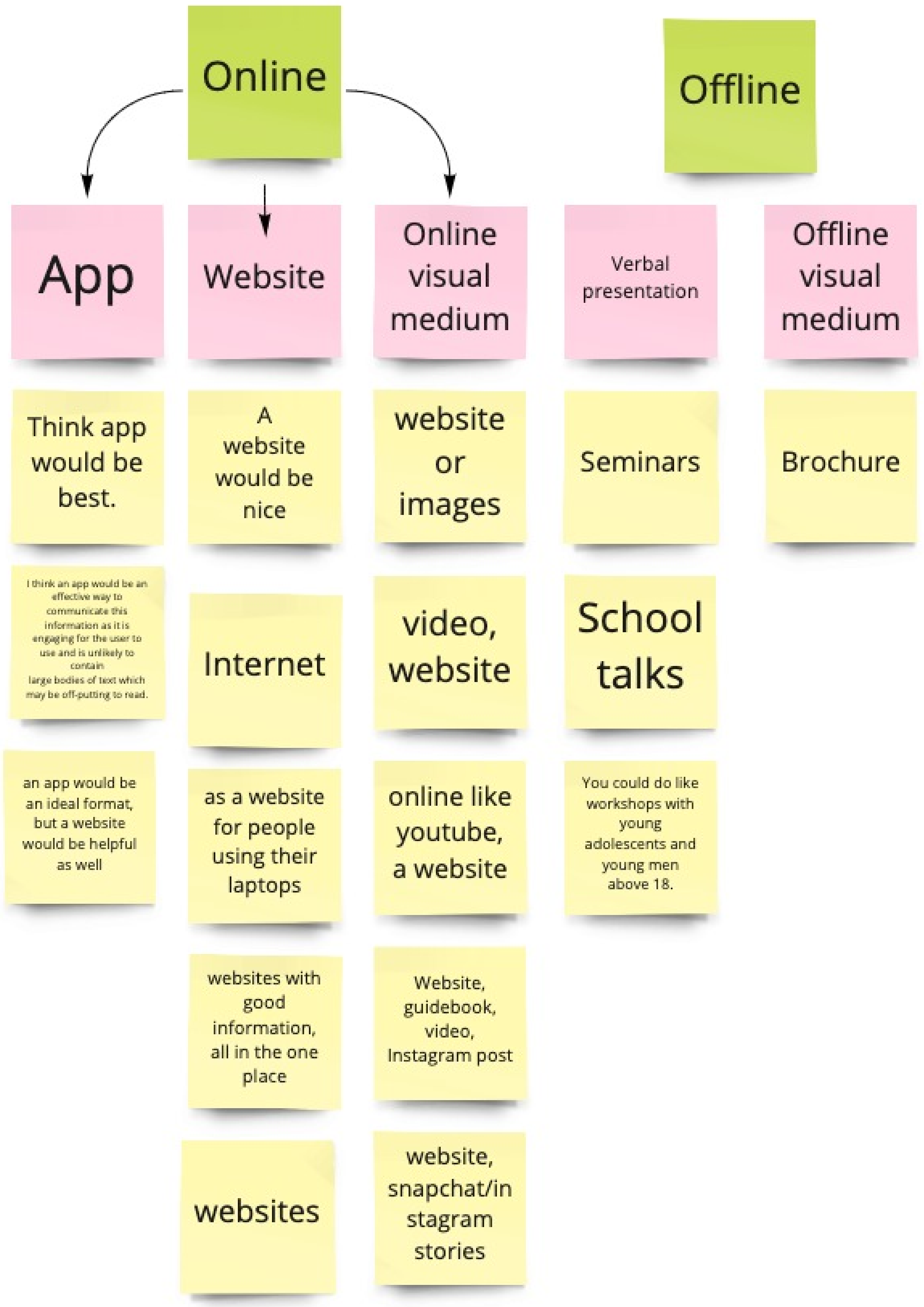

Online
Offline
App
Website
Online visual medium
Verbal presentation
Offline visual medium
Think app would be best.
A website would be nice
website or images
Seminars
Brochure
I think an app would be an effective way to communicate this information as it is engaging for the user to use and is unlikely to contain large bodies of text which may be off-putting to read.
Internet
video, website
School talks
an app would be an ideal format, but a website would be helpful as well
as a website for people using their laptops
online like youtube, a website
You could do like workshops with young adolescents and young men above 18.
websites with good information, all in the one place
Website, guidebook, video, Instagram post
websites
website, snapchat/instagram stories
miro
