# Supplementary material for: Development of a Peer Support Mobile App and Web-Based Lesson for Adolescent Mental Health (Mind Your Mate): User-Centered Design Approach
Source: JMIR Form Res. 2022 May 27;6(5):e36068. doi: 10.2196/36068 (PMC9187963; doi:10.2196/36068)
Supplement: Multimedia Appendix 4 [file formative_v6i5e36068_app4.docx]

# Multimedia Appendix 4

| **Themes from open ended survey questions thematic analysis.** | | |
| --- | --- | --- |
| Question | Theme | Examples |
| Favourite aspects of regularly used apps | Socialising | - “My favourite things about these apps is that you able to check in on your mates and see how they are going and what they are doing.” - “I love TikTok because I understand the humour and it is easy digestible content that makes me laugh. The community is unlike other and is very funny content….I like messenger cause I can talk to my friends” |
|  | Intuitive use | - “How simple and straight forward they are. The fact the app is free and I can use it with my friends.” - “The accessibility that they give you to others and online resources, how smooth and well developed the technology is.” - “Easy to use.” |
|  | Enjoyable | - “They provide a wide array of content, including entertainment (first four apps), fun education (from YouTube), and convenience (WhatsApp messaging and Bible app).” - “Watch others' lives, videos about games and sports and stuff.” |
| Annoying things experienced when using those apps | Interruptions to use | - “Glitches out, music doesn't download, new look updates.” - “Ads, needing to pay money, functions don't work unless you pay” - “Sometimes it lags or glitches which can be annoying to fix.” |
|  | Requires resources | - “They are addicting therefore using up my data.” - “My phone battery drains very quickly when using TikTok and the battery life has decreased” |
|  | Uncertainty in use | - “They are distractions sometimes” - “Sometimes these apps can provide us with unnecessary information. Also, some apps take longer and are harder to use.” |
| What would have been helpful in a time you were worried about a friend | Third party person | - “Another person knowing what I knew about that friend.” - “Seeing a counsellor to get professional help/analysis on the friend's issue based on their symptoms and actions.” - “Someone telling me that it isn't always entirely my responsibility to help them and being able to reach out to other people who have more experience or knowledge than me and are probably better eqquiped to help them.” |
|  | Best ways to help | - “Basic methods on how to help them.” - “Reaching out and asking whether they were okay or not, and if there was anything i could do to help them move through it.” |
|  | How to have a conversation | - “It would be helpful if someone could tell me how I should approach my friend because I don't really know whether if I will say something that may trigger something and make the situation worse.” - “To know helpful questions and ways to talk to them.” - “If I had known different ways to comfort them or be able to have the knowledge on what I can do” |
| What kind of information | How to have a conversation | - “How long they have started to use alcohol or drugs. How did they first start, was it peer pressure - Why do they use alcohol and drugs and for what reasoning?” - “What are the reasons they are using drugs or alcohol. Are they being influenced” |
|  | Information to discourage use | - “Evidence and statistics to look at the situation logically - e.g. The associated health risks, side effects, deaths from substance abuse, any potential benefits…” - “How to discourage them and remind them of the consequences.” |
|  | Third party support | - “How to help, treat them and help them if their drinking is not drastically out of control. Also, who to contact if it is drastically out of control.” - “Where to go, who to tell, what to tell them” |
| Other formats for this information (other than app) | Website | - “As a website for people using their laptops” - “Websites with good information, all in the one place” |
|  | App | - “I think an app would be an effective way to communicate this information as it is engaging for the user to use and is unlikely to contain large bodies of text which may be off-putting to read.” - “An app would be an ideal format” - “Think app would be best.” |
|  | Offline visual medium | - “Brochure” |
|  | Online visual medium | - "Snapchat/Instagram stories” - “Online like YouTube” - “Video, Instagram post” |
|  | Verbal presentation | - “You could do like workshops with young adolescents and young men above 18.” - “Seminars” - “School talks” |
