# Supplementary material for: Development of a Peer Support Mobile App and Web-Based Lesson for Adolescent Mental Health (Mind Your Mate): User-Centered Design Approach
Source: JMIR Form Res. 2022 May 27;6(5):e36068. doi: 10.2196/36068 (PMC9187963; doi:10.2196/36068)
Supplement: Multimedia Appendix 5 [file formative_v6i5e36068_app5.docx]

# Multimedia Appendix 5: Logic model for the *Mind your Mate* program

**AUDIENCE**

**INPUTS**

**OUTCOMES**

**OUTPUTS**

**MECHANISMS**

**SITUATION**

*AOD = Alcohol and other drug

One classroom lesson (40 mins)

Smartphone app

**ACTIVITIES**

Adolescents aged 15-16 years old

Year 9/10 students

**THREATS / EXTERNAL FACTORS**

Lack of engagement with the app or classroom lesson, lack of time to implement classroom lesson, lack of internet connection, low smartphone ownership, technological issues, stigma surrounding mental health & AOD issues, influence of the social environment

**Medium-term**

Reduced mental health symptoms

Reduced AOD uptake

**Short-term**

Increased mental health, AOD literacy

Increased intentions to seek help if needed

**Long-term**

Establish good mental health & help-seeking habits in adolescence

# of lessons completed

# of downloads of the app

# of modules skill completed within the app

# of interactive app elements used (e.g. self-care mood tracking, conversations scheduled with friends)

Increase levels of adolescents’ mental health & AOD literacy

Equip students with active listening skills and information about support options

Self-care skills

Normative education: challenging misconceptions about peer substance use & reduce stigma

Provide harm-minimisation strategies to stay safe & seek help

Adolescents don’t seek professional help for mental health or AOD related problems. Adolescents are frequently supporting friends with early mental health or AOD concerns, however are not equipped with relevant skills and referral options. If not prevented or treated these problems can have negative impacts later in adulthood.

- Time
- School staff
- Access to internet & smartphone
- Research
